# Development of Polyvinylidene Fluoride Membrane via Assembly of Tannic Acid and Polyvinylpyrrolidone for Filtration of Oil/Water Emulsion

**DOI:** 10.3390/polym13060976

**Published:** 2021-03-22

**Authors:** Normi Izati Mat Nawi, Syasya Ong Amat, Muhammad Roil Bilad, Nik Abdul Hadi Md Nordin, Norazanita Shamsuddin, Saiful Prayogi, Thanitporn Narkkun, Kajornsak Faungnawakij

**Affiliations:** 1 Department of Chemical Engineering, Universiti Teknologi PETRONAS, Bandar Seri Iskandar 32610, Malaysia; normi_16000457@utp.edu.my (N.I.M.N.); syasya.ong@gmail.com (S.O.A.); 2 Faculty of Applied Science and Technology, Universitas Pendidikan Mandalika (UNDIKMA), Jl. Pemuda No. 59A, Mataram 83126, Indonesia; saifulprayogi@ikipmataram.ac.id; 3 Faculty of Integrated Technologies, Universiti Brunei Darussalam, Jalan Tungku Link, Bandar Seri Begawan BE1410, Brunei; norazanita.shamsudin@ubd.edu.bn; 4 National Nanotechnology Center (NANOTEC), National Science and Technology Development Agency (NSTDA), 111 Thailand Science Park, Pathum Thani 12120, Thailand; thanitporn.nar@ncr.nstda.or.th (T.N.); kajornsak@nanotec.or.th (K.F.)

**Keywords:** membrane fouling, tannic acid, hydrophilic additives, polyvinylidene fluoride, polyvinylpyrrolidone

## Abstract

Wastewater containing oil/water emulsion has a serious ecological impact and threatens human health. The impact worsens as its volume increases. Oil/water emulsion needs to be treated before it is discharged or reused again for processing. A membrane-based process is considered attractive in effectively treating oil/water emulsion, but progress has been dampened by the membrane fouling issue. The objective of this study is to develop polyvinylidene fluoride (PVDF) membranes customized for oil/water emulsion separation by incorporating assembly of tannic acid (TA) and polyvinylpyrrolidone (PVP) in the polymer matrix. The results show that the assembly of TA/PVP complexation was achieved as observed from the change in colour during the phase inversion and as also proven from the characterization analyses. Incorporation of the TA/PVP assembly leads to enhanced surface hydrophilicity by lowering the contact angle from 82° to 47°. In situ assembly of the TA/PVP complex also leads to enhanced clean water permeability by a factor of four as a result of enhanced mean flow pore size from 0.2 to 0.9 µm. Owing to enhanced surface chemistry and structural advantages, the optimum hydrophilic PVDF/TA/PVP membrane poses permeability of 540.18 L/(m^2^ h bar) for oil/water emulsion filtration, three times higher than the pristine PVDF membrane used as the reference.

## 1. Introduction

The rapid development of industries such as palm oil mill, refinery plants and crude oil production largely increases the volume of discharged oily wastewater, including wastewater in the form of emulsion. This kind of wastewater must be properly treated before being discharged to the environment or reused again for processing since direct discharge to the environment pollutes water bodies, impairs drinking water and groundwater resources, and endangers aquatic livings and human health. Oil/water emulsion is constituted of a homogenous and stable system of immiscible liquids of water and oil [1]. An emulsion is very stable since it has low interfacial tensions (1 to 7 mN/m) under the presence of emulsifiers such as surfactants [2]. Thus, the high stability of the oil/water emulsion system makes it very difficult to be separated [3]. Membrane-based processes have been recognised as promising methods in effective separation of oil/water emulsion. They are very stable and robust, especially in handling oil/water emulsion containing droplets with sizes below 20 μm [4] at an oil concentration of <1000 ppm. This technology uses the principle of size exclusion in which the small pores of the membrane restrict the permeation of oil droplets through the pore, but selectively allow water to pass through. Membrane-based processes also have great separation performance, rendering them as better options in treating oil/water emulsion. Some of the advantages include a high rate of waste removal, environmentally friendly, simple design and operation and easy to integrate with other processes via process intensification [5,6,7,8]. However, membrane separation efficiency reduced over time due to membrane fouling, which substantially diminishes its long-term performance.

Polyvinylidene fluoride (PVDF) polymer is one of the most popular materials used for the fabrication of membrane due to its good mechanical properties, chemical stability and excellent thermal resistance [9]. However, PVDF polymer is hydrophobic with a typical contact angle of above 90° [10], thus filtration of oil/water emulsion using a plain PVDF membrane results in low permeability and vulnerability from membrane fouling. Oil droplets in oil/water emulsion easily foul plain PVDF membrane since oil droplets can strongly adhere to the membrane surface [10]. Fortunately, PVDF-based membrane can be made hydrophilic by incorporating additives during membrane fabrication to overcome its high membrane fouling propensity [11,12]. For instance, Cui et al. [4] reported a biomimetic surface modification by virtue of the polydopamine anchored by silicone dioxide on the PVDF membrane by enzymatic oxidation. The fabricated membrane achieved high oil/water emulsion permeability of 572 L/(m^2^ h bar). Furthermore, Zhu et al. [13] developed a hydrophilic PVDF membrane aided with a zwitterionic polymer precursor as the additives in the membrane fabrication. In situ crosslinking was used to immobilize the additives and a subsequent sulfonation reaction on the membrane surface to ensure long-term stability of the fabricated membrane. The membrane exhibited excellent initial oil/water permeability of 6350 L/(m^2^ h bar) but dropped by about 90% after 10 h of filtration. Recently, the vapor-induced phase separation technique in the presence of polyethylene glycol additive had also been applied to develop polysulfone-based membrane for oil/water emulsion filtration [14]. The results reveal that increasing the exposure time before immersion from 0 to 60 s improves the clean water permeability by 52.7% and reduces the membrane surface water contact angle from 70.3° to 57.7°. Recently, Cardea et al. (2018) reported supercritical-assisted processes to develop porous PVDF polymer, suggesting the versatility of options for the production of PVDF membrane [15].

Aside from developing membrane material for treating industrial wastewater, recent research has also addressed the necessity to investigate on-demand oily wastewater treatment [16,17,18]. Yan et al. [19] integrated carbon nanotubes (CNTs) and titanium dioxide nanoparticles (TiO_2_ NPs) to prepare composite membrane applied for on-demand oil/water separation. The optimum membrane showed excellent separation performance with a flux up to 40,000 L/(m^2^ h bar) for water-in-dichloromethane emulsion, and separation efficiency above 98.89%. In another study, a low-cost and durable flexible membrane made of layered double hydroxide nanosheet on cellulose support was reported [20]. The membranes not only show high separation efficiency (>94.4%), great chemical durability and good recyclability, but also display high separation efficiency for surfactant-stabilized water-in-oil emulsions and good flux (500 L/(m^2^ h bar)). 

Some researchers have explored tannic acid (TA) as a hydrophilic additive due to its desirable binding ability on various substrates, nontoxicity properties and low cost [21]. For example, Wu et al. [22] developed a hydrophilic PVDF-based membrane with good antifouling capability by coating with 3-chloropropylrimethoxysilane/polyethyleneimine and TA. The additives incorporate metal ions to ensure a more stable membrane was formed. The fabricated membrane achieves a good oil/water emulsion permeation flux of about 8000 m^3^/m^2^hbar with oil rejection of >99.6%. Ong et al. [23] also reported a similar method of fabrication, which is via coating with TA to obtain hydrophilic PVDF-based membrane. The method employed rapid deposition of TA on the PVDF membrane surface followed by oxidation with sodium periodite (NaIO_4_). The TA is then bound to the PVDF polymer through Van der Waals force and hydrogen bonding. The fabricated membrane shows a good oil/water mixture filtration with water flux of 401 ± 97 L/(m^2^ h).

Generally, the most common method to alter the structure and surface properties of the resulting membrane is by incorporation of hydrophilic additives into the polymer solution [23]. Most of the resulting PVDF membranes have a high hydrophilic properties and excellent rejection performance. However, some drawbacks are identified, namely high time consumption, usage of harsh chemicals and unstable coating on the membrane surface. For example, the surface grafting method has effective additive stability but a complex fabrication process [24]. Moreover, the surface coating method is relatively simple but has rather poor instability. Thus, phase inversion is one of the most widely used methods for PVDF membrane fabrication due to its simplicity and fast process [25]. Despite its advantages, the blending of hydrophilic additives using this method suffers from a high tendency of additive leaching (washed away) during fabrication and filtration [10,26]; as such, its impact on enhancing hydrophilicity is largely reduced, resulting in a hydrophobic membrane. The additives have high affinity to water (typically used as a non-solvent in the phase inversion), promoting the mobility of the additive towards the water phase. 

In this study, the leaching problem of the hydrophilic additives is limited by introducing complexation of TA and polyvinylpyrrolidone (PVP) from the dope solution, which forms an assembly facilitated by the Fe^3+^ present in the non-solvent bath. The assembly of TA/PVP is expected through the formation of hydrogen bonding and provides binding sites for metal ions. The formation of TA/PVP/Fe^3+^ assembly via complexation reaction lowers their mobility and limits it from leaching out from the membrane matrix. The resulting membrane is then expected to pose hydrophilicity and antifouling propensity for a good oil/water emulsion separation. Hence, four types of PVDF membranes were fabricated to investigate the effect of incorporating the assembly of TA/PVP in the membrane matrix via complexation method during the fabrication process. The properties and the hydraulic performance of the developed membranes were evaluated. 

## 2. Materials and Methods

### 2.1. Membrane Preparation

**Figure 1:**
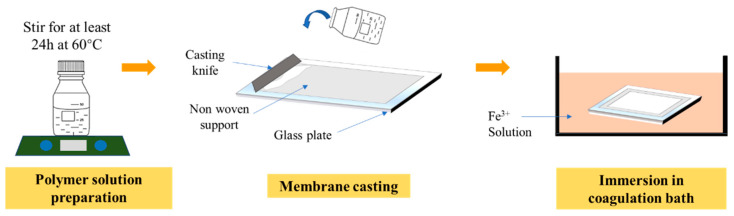
Illustration of step by step of membrane preparation through the phase inversion process.

In this study, four membranes were prepared. PVDF (average M_w_ ~534,000 by GPC, Sigma Aldrich, St Louis, MO, USA) was used as the polymer, dimethylacetamide (DMAC, Sigma Aldrich, St Louis, MO, USA) as the solvent, tannic acid (TA, Sigma Aldrich, St Louis, MO, USA) and PVP (average M_w_ ~10,000) as the additives and iron (III) chloride hexahydrate (FeCl_3_6H_2_O, Sigma Aldrich, St Louis, MO, USA) as the non-solvent. Table 1 summarizes the composition of the dope solutions for membrane fabrication. All of the chemicals (PVDF, TA, PVP and DMAC) were mixed in a Schott bottle and stirred for at least 24 h or until homogenously mixed at 60 °C. After the dope solution was homogenously mixed, it was left idle overnight to eliminate air bubbles to avoid the thin film defect. As shown in Figure 1, the solution was casted using a casting knife with a wet thickness of 200 µm on a non-woven support on top of a glass plate. Then, the casted film was immediately immersed in a coagulation bath containing predetermined concentration of iron (III) chloride hexahydrate (as shown in Table 1) for at least 24 h to completely remove the solvent trace from the membrane matrix. The membrane was kept wet until usage. 

### 2.2. Feed Preparation

The synthetic oil/water emulsion feeds with oil concentrations of 1000 ppm were prepared by using sodium dodecyl sulfate (SDS, 98% purity, Sigma Aldrich, St Louis, MO, USA), real crude oil (obtained from a crude oil well in Malaysia), and distilled water as the surfactant, the oil, and the water, respectively. For each oil concentration, a 1:99 *w*/*w* SDS to oil ratio was first prepared and used to form stable emulsions. The prepared oil/SDS mixture was mixed with one litre of water separately at a stirring rate of 3500 rpm for 24 h to obtain the desired feed solution of 1000 ppm oil/water emulsion. A small volume of feed samples was later analysed using a particle size and zeta potential analyser (Malvern, Zetasizer Nano ZSP) to map the oil droplet size distribution. The oil droplet distribution shows multimodal at sizes of 0.25, 1 and 4 µm.

### 2.3. Membrane Characterization

The properties of the resulting membranes were determined with respect to membrane morphology, pore size distribution, surface hydrophilicity, chemical surface distribution and clean water permeability (CWP). For the membrane morphological study, scanning electron microscopy (SEM, FEI Quanta-250, Thermo Fisher Scientific, Waltham, MA, USA) was used to obtain the surface and cross-section images of the resulting membranes. Prior to testing, the samples were immersed in liquid nitrogen to freeze-fracture the sample and then were coated with a thin gold layer. Furthermore, a capillary flow porometer (CFP, Porolux 1000, Berlin, Germany) was used to determine the pore size distribution (PSD) of the membranes, while the membrane surface’s hydrophilicity was evaluated using a goniometer (Ramé-Hart 260, Succasunna, NJ, USA) to obtain the membrane surface’s contact angle. For the chemical surface distribution, EDX mapping was performed based the SEM image of membrane surface. At the same time, Fourier transform infrared spectroscopy (FTIR, PerkinElmer Inc., Waltham, MA, USA) with spectra ranging from 400 to 4000 cm^−1^ and an X-ray photoelectron spectrometer (XPS, K-Alpha^TM^, Thermo Scientific, Waltham, MA, USA) were used to determine the chemical composition of the membrane surface. 

In addition, tests for water uptake (*WU*, %) and swelling degree (*SD*, %) of the resulting membranes were performed. Water uptake was measured by immersing membrane strips with a dimension of approximately 30 × 10 mm in deionized water at 25 °C for 24 h. Then, the membrane strips were removed from the water bath and the surface was carefully dried using absorbent paper. Equation (1) was used to calculate the water uptake of the membrane.

(1) $$WU=\;\frac{m_{hydrated}-\;m_{dry}}{m_{dry}}\;\times \;100\%$$

Meanwhile, swelling degree was measured by comparing the dimensions of membrane strips before and after immersion in water. In this study, membrane thickness has been utilized to calculate the swelling degree according to Equation (2):

(2) $$SD=\;\frac{x_{hydrated}-\;x_{dry}}{x_{dry}}\;\times \;100\%$$

where $m_{dry}$ is the mass of the membrane strips before being immersed, $m_{hydrated}$ is the mass of the strip after immersion, $x_{dry}$ is the membrane thickness before and $x_{hydrated}$ is the thickness after immersion. 

### 2.4. Membrane Filtration Test Module Set-Up

**Figure 2:**
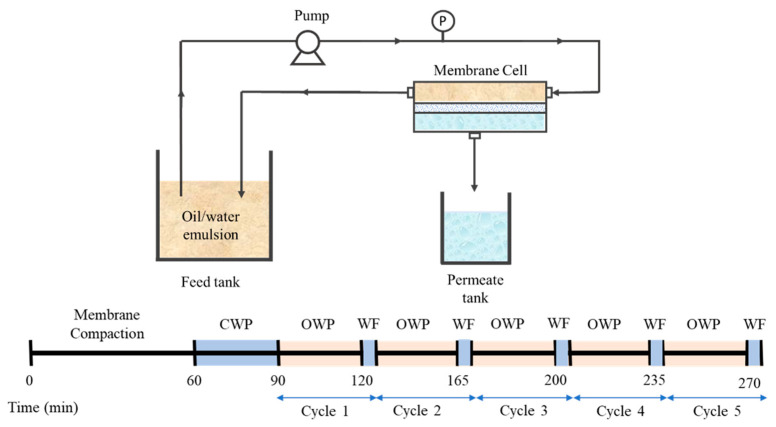
Illustration of the crossflow filtration setup.

Figure 2 illustrates the crossflow microfiltration setup that was used to evaluate the hydraulic performance of the developed membranes. The filtration was operated at a fixed pressure difference of 0.2 bar with effective membrane area of 22.4 cm^2^. The filtration was conducted continuously starting with 60 min of membrane compaction, followed by 30 min of CWP test. The filtration was then proceeded with 30 min of oil/water emulsion (OWF) followed by five minutes of water flushing (WF) which was considered as filtration cycle 1. It was followed by another four filtration cycles. For each filtration cycle, the permeate volume was measured every 5 min to avoid significant changes to the concentration of the feed. 

The volume of permeate produced for every filtration was collected and measured before being returned to the feed tank. Membrane permeability (*L*, L/(m^2^ h bar))was calculated using Equation (3), while the percentage of oil rejection (*R*, %) of the filtration test was determined using Equation (4):

(3) $$L\;=\;\frac{∆V}{∆t\;A\;∆P}$$

(4) $$R=\;\frac{C_{0,feed}\;-\;C_{0,permeate}}{C_{0,feed}}\;\times 100\;\;\;\;\;\;$$

where ∆*V* is the collected permeate volume (L), ∆*t* the filtration time (h), *A* the membrane effective area (m^2^), ∆*P* the transmembrane pressure (bar), *C*_0*,feed*_ (mg/L) the concentration of oil in the feed and *C*_0*,permeate*_ (mg/L) the concentration of oil in the permeate. 

### 2.5. Membrane Fouling Analysis

To evaluate the fouling properties of the developed membranes, the evolution of permeability over the five cycles of filtration was analysed. The fouling was categorized into total fouling ($F_{T}$), reversible fouling ($F_{R}$) and irreversible fouling ($F_{I}$). Total fouling was defined as the amount of permeability loss (%) after completing oil/water emulsion filtration. Reversible fouling was defined as the percentage of permeability which could be recovered through water flushing. Irreversible fouling was defined as the percentage of permeability which cannot be recovered by water flushing. Equation (5) shows the calculation for total fouling, where $L_{,\mathrm{Cwp}}$ is the permeability of clean water filtration and $L_{,o/w}$ is the permeability of oil water filtration of the membrane.

(5) $$F_{T}=\frac{L_{,\mathrm{Cwp}}-L_{,o/w}}{L_{,\mathrm{Cwp}}}\;\times \;100\%\;$$

The reversible fouling was calculated using Equation (6), where $L_{,w}$ is the permeability of clean water flushing of the membranes.

(6) $$F_{R}=\frac{L_{,w}-L_{,o/w}}{L_{,\mathrm{Cwp}}}\;\times \;100\%\;$$

Then, the reversible fouling was calculated by subtracting total fouling and reversible fouling, as shown in Equation (7).

(7) $$F_{I}=F_{T}-F_{R}\;\;$$

## 3. Results and Discussion

### 3.1. Membrane Properties

#### 3.1.1. Morphology

**Figure 3:**
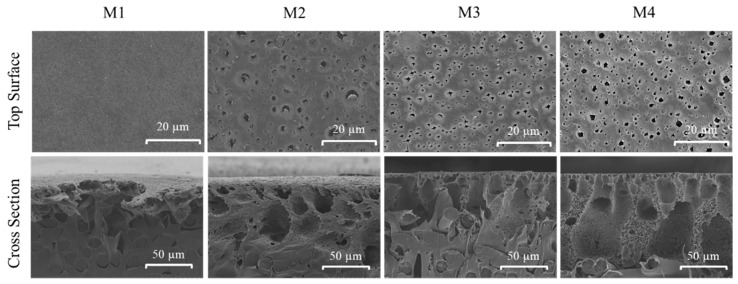
The top surface and cross-sectional morphologies of the developed membranes.

The surface morphologies of each membrane were studied through SEM images. SEM analysis was performed to study the morphological changes as a result of additive dosing as well as from the effect of Fe^3+^ in the non-solvent bath during membrane fabrication. Figure 3 shows that the pristine PVDF membrane (M1) has a smooth surface with homogenous spatial distribution of surface pores. The addition of TA/PVP additives demonstrates obvious changes in the amount and size of the surface pores on the membrane surface. In comparison to M1, the surface pore size (pore mouth) of M2, M3 and M4 slightly increases, suggesting the efficiency of TA and PVP additives in altering the mechanism of membrane formation. Both TA and PVP are expected to be partly incorporated into the PVDF matrix.

When comparing M3 and M4, the presence of Fe^3+^ in the non-solvent bath results in significant changes in the surface morphology, in which the sizes of the pore mouths are smaller than the ones in M2 as well as showing a more homogenous distribution of the pore mouth on the membrane surface. This can be explained by the ability of polyphenol in TA to impart the binding sites to form a crosslinking hydrogen bond reaction with PVP and Fe^3+^ that alters the formation mechanism of the membrane, affecting the surface morphology [27]. Metal ions such as Fe^3+^ also have a high oxidation state which could induce the polymerization rate of TA [22], resulting in a more prominent impact on the mechanism of membrane formation. 

The impact of the TA/PVP additive in enhancing the porosity of the membrane is also obvious from the porosity data of M1, which are significantly lower than the rest. The porosity data of M1, M2, M3 and M4 are 13.5, 40.8, 42.1 and 44.9%, respectively. The high membrane porosity is translated into the formation of a thicker membrane as also confirmed by the thickness data of M1, M2, M3 and M4 of 202.3, 235.3, 242.7 and 259.0 µm, respectively. 

Figure 3 also shows the SEM images of cross-sectional morphologies and structures of the prepared membranes. All of the TA/PVP containing membranes (M2, M3 and M4) show a denser skin layer indicating the TA/PVP impacts on altering the resulting membrane structure. The presence of TA/PVP additives in the modified membranes is well known to act as pore performing agents [28], but may be partly leached out during the phase separation process, also affecting the final membrane morphology [29], which is further analysed based on chemical analysis of the membrane samples. 

#### 3.1.2. Pore Size and Distribution

**Figure 4:**
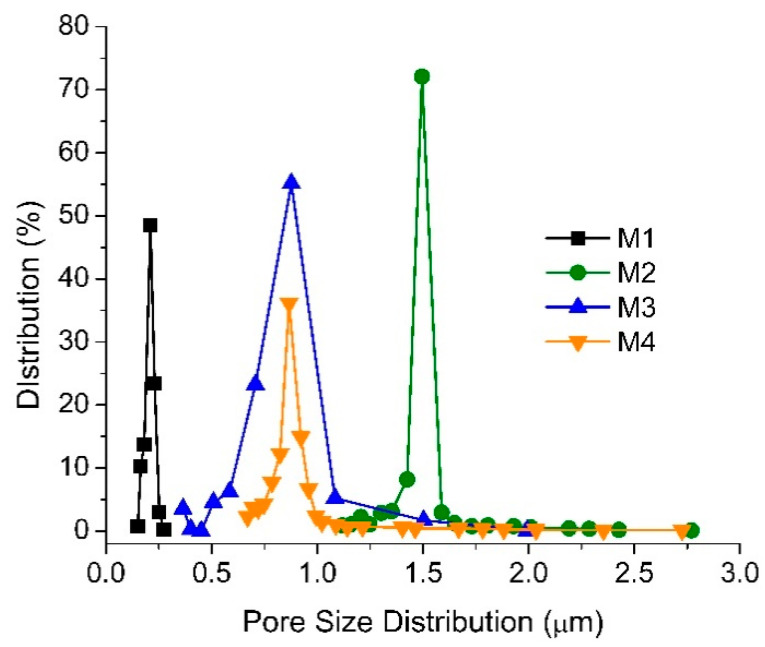
The pore size distribution of the membrane samples.

Figure 4 shows the pore size distribution, suggesting that the addition of TA/PVP into the dope solution of M2, M3 and M4 increases the mean pore sizes, respectively to 1.54, 1.00 and 0.90 µm compared to the 0.22 µm mean pore size of the M1. The results are in line with the morphological images in Figure 3 where the addition of PVP additives as a pore former significantly affects the structure of the membrane. During the phase inversion, the PVP/TA present in the dope solution seems to migrate toward the polymer-lean phase or the bulk non-solvent bath due to its good affinity with water, allowing the formation of more pores in the PVDF matrix. The larger pore size of M2 can be ascribed by the merging of smaller pores [30] or due to its relatively free mobility, unlike the assembly of TA/PVP that poses larger steric hindrance. 

The loading of additive in M2 changes both dope solution viscosity and stability. The former leads to a slower rate of demixing, while the latter accelerates demixing during the phase inversion process. Judging from the morphology and the surface pore size, the result of the TA/PVP loading is an increase in the demixing rate, leading to the formation of a larger pore size and more porous morphology. The increase in demixing can be attributed to the migration of the hydrophilic TA and PVP to the polymer-lean phase, resulting in a large mean flow pore size as shown in Figure 4. The smaller mean flow pore sizes of M3 and M4 compared to M2 justify the ability of TA to complex with PVP and Fe^3+^ forming a large superstructure with low diffusivity/mobility due to steric hindrance in the highly viscous polymer-rich phase, which eventually results in membranes with smaller pore size. The range of pore size distribution of M2 and M3 is as large as of M2, suggesting pre-migration of a fraction of PVP prior to forming the TA/PVP/Fe^3+^ complex. M4 showing a higher percentage of pore distribution at a smaller pore size region suggests a higher yield of TA/PVP/Fe^3+^ complexation, as also supported by the membrane thickness data (Section 3.1.1). 

#### 3.1.3. FTIR Spectra

**Figure 5:**
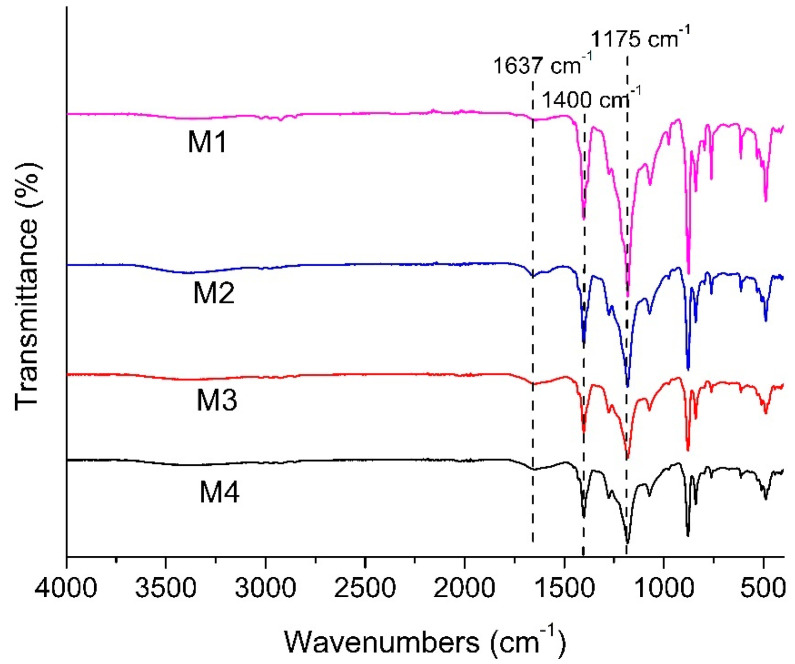
FTIR spectra of the developed membranes.

Figure 5 shows the FTIR spectra of all membranes, detailing the chemical functional groups available nearby the membranes surface. Each membrane shows a peak at 1400 and 1175 cm^−1^, showing the C-H bending vibration and C-F stretching absorption from the PVDF polymer chain. New peak absorption is observed at 1637 cm^−1^ for the modified membranes (M2, M3 and M4), which is attributed to C=O stretching vibration originating from the complex of the polyphenol group of TA. 

**Figure 6:**
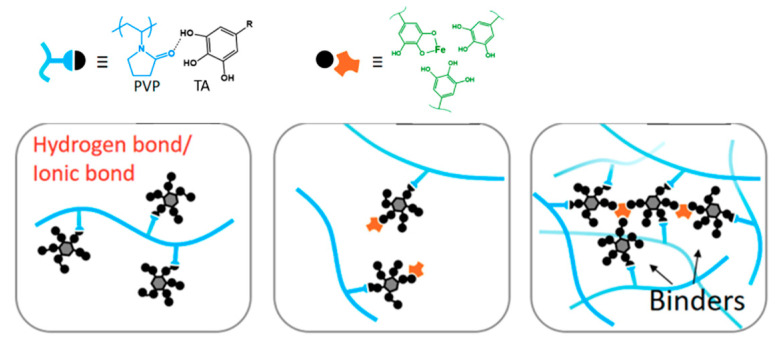
Illustration of supramolecular formation based on PVP/TA/Fe^3+^.

The intensities of the peaks within a sample relative to the corresponding peaks from another sample indicate the relative amount of component residing in the membrane matrix. A lower intensity of peaks at 1400 and 1175 cm^−1^ for M4 relative to M1 corresponds to the lower fraction of functional groups from the PVDF polymer due to the intensities of other peaks attributed to the additive’s presence in M4. Greater intensity at 1637 cm^−1^ relative to M2 indicates a higher amount of TA/PVP/Fe^3+^ complex in the membrane matrix. The findings suggest that complexation of PVP/TA/Fe^3+^ enhances their retention in the matrix by limiting the leaching out from the cast film during the phase inversion process [27]. According to Fan et al. (2017) [27], the formation mechanism of the three component TA/PVP/Fe^3+^ complex follows the following scheme (Figure 6). TA works as binders to functionalize the PVP chains via hydrogen bonds or ionic bonds. TA binds PVP together with the aid of Fe^3+^. Multiplex coordination results in the cross-linking of TA-connected PVP chains. The presence of the complex is then expected to enhance the antifouling property of the membrane thanks to functional groups that can form hydrogen bonding with water.

#### 3.1.4. Surface Elemental Composition

**Figure 7:**
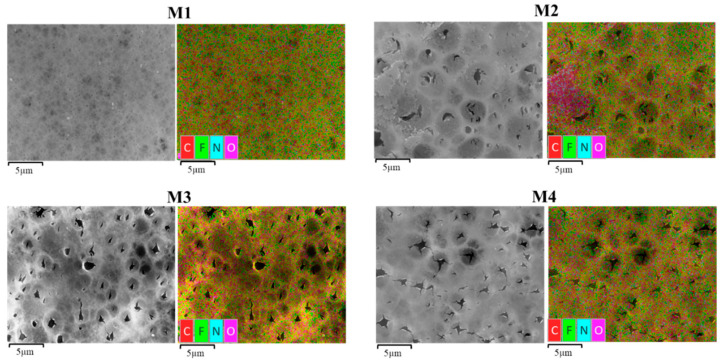
EDX mapping of each membrane.

To further confirm the preservation of the PVP/TA in the modified membranes, the energy-dispersive X-ray spectroscopy (EDX) mapping of elements on the membrane surface are shown in Figure 7. The results indicate the presence of carbon (C), fluoride (F) and oxygen (O) in each membrane at different compositions (Table 2). Compared to the pristine PVDF membrane, increasing percentages of oxygen element are observed on the modified membrane M2, M3 and M4, which justifies the successful incorporation of TA additives into the membrane matrix. From Ong et al. [23], the large increase in O in the modified membranes was from TA, which is very rich in O element content. 

Figure 7 shows that the distribution of C, F and O elements in M1 had a significant change compared to the rest. The relative amount of C and F is significantly reduced, while the abundance of N and O elements increases. The increasing relative content of the O and N elements supports the previous suggestion of higher retention of TA/PVP in the modified membrane matrix, in which the increase in N element is attributed to the presence of the PVP [10]. A good distribution of the elements shown in Figure 7 indicates formation of homogeneous membrane structure.

**Figure 8:**
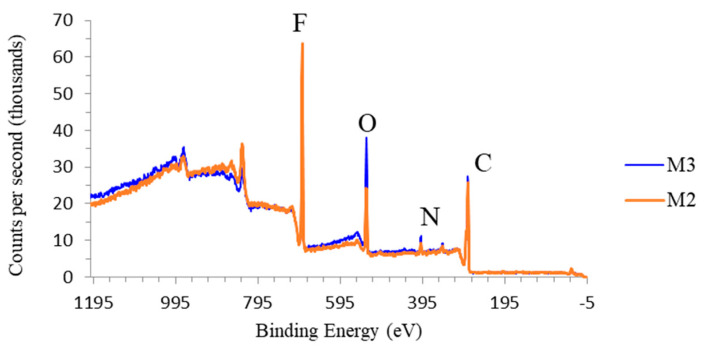
XPS wide scan spectra of M2 and M3 showing the higher additive retention due to complexation reaction of TA/PVP/Fe^3+^.

To further confirm the effect of TA/PVP/Fe^3+^ complexation in enhancing their retention in the PVDF matrix, XPS analysis was applied to obtain the elemental composition of M2 and M3. Since no Fe^3+^ was included during M2 fabrication, a higher degree of additives leaching is expected. The dope solution compositions for both membranes were identical, but the non-solvent of M3 contained Fe^3+^ to induce TA/PVP complexation. As shown in Figure 8 and Table 3, the O and N elements peak are observed for both membrane M3 and M2 indicating the presence of the residual additive of TA and PVP, supporting the data obtained from EDX (Figure 7). However, M3 has a higher oxygen peak showing more O element compared to M2, which confirms the higher retention of additive via complexation. A substantially much higher composition of O element in M3 suggests that the presence of Fe^3+^ in the coagulation bath incorporates more TA and PVP by forming a stable complex [31]. The large complex of PVP/TA/Fe^3+^ resulted in poor particle mobility during the solvent/non-solvent exchange, which was then entrapped in the matrix.

#### 3.1.5. Surface Contact Angle

**Figure 9:**
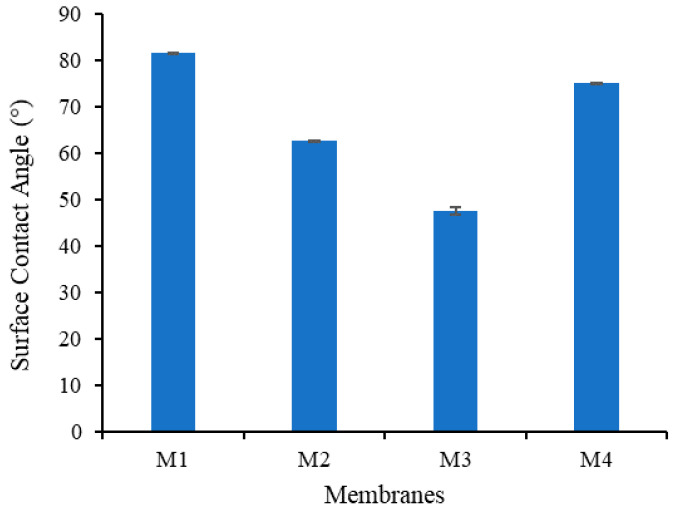
Static water contact angle on the membrane surface.

The influence of the additives on the water contact angle (WCA) of the membrane is shown in Figure 9. Addition of TA and PVP to the polymer solution has a significant impact on the membrane composition, thus affecting the membrane hydrophilicity properties [25]. Surface WCA is positively associated with the surface hydrophilicity. The WCA for M1 is 81.59, and decreases to 62.81, 49.01 and 75.07 for M2, M3 and M4, respectively, indicating the improvement in the hydrophilicity for the modified membranes. The presence of polar functional groups from TA and PVP formed a hydrophilic layer on the membrane wall, which provides the hydrogen bonding sites for water [32]. 

The ability to form a coordination reaction between TA and Fe^3+^ results in the lowest WCA for M3. However, higher concentration of Fe^3+^ solution used in the non-solvent for preparation of M4 shows only a small decrement in the WCA compared to M1. It is speculated that a higher concentration of Fe^3+^ enhances the amount of TA/PVP assembly but they reside in the inner layer of the membrane matrix. The formation of a large complex results in a very low mobility toward the surface and has low impact in lowering the WCA. Alternatively, a high concentration of Fe^3+^ in the non-solvent solution resulted in complex TA-Fe^3+^ aggregation on the membrane wall, which increased the surface roughness, promoting high WCA for M4 as proposed elsewhere [33]. 

#### 3.1.6. Clean Water Permeability

**Figure 10:**
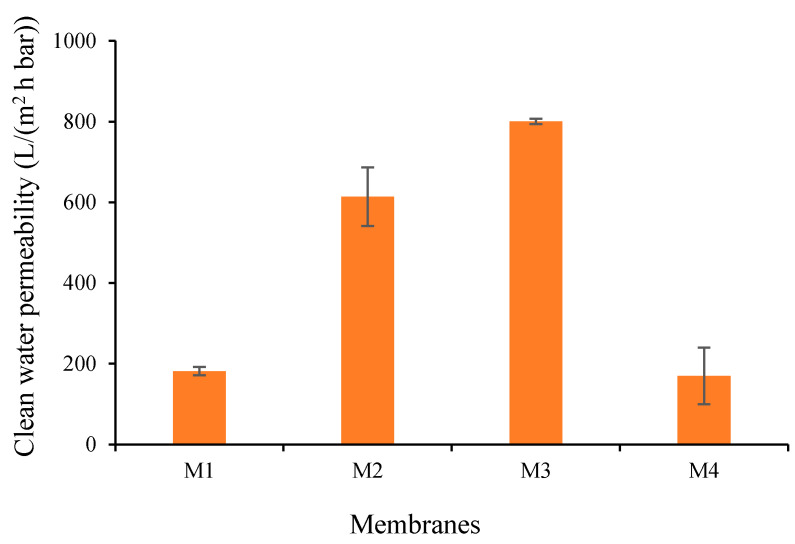
Clean water permeability of each membrane.

To further justify the impact of additives and their complexation on the intrinsic membrane resistance, CWP of all prepared membranes were measured (Figure 10). When compared to the M1 with CWP of 181.55 L/(m^2^ h bar), the CWPs of M2 and M3 increase to 613.84 and 800.6 L/(m^2^ h bar), respectively. The increment of the CWP can be attributed to improved hydrophilicity properties (low WCA as in Figure 9) and the increasing pore size (see Figure 4), in line with the literature [34]. The increase in the hydrophilicity increases the wettability of the membrane due to improvement in the interaction between the membrane layer with the water molecule, thus contributing to the higher CWP [35]. Therefore, higher hydrophilicity of M3 compared to M2 overshadowed the impact of pore size (Figure 4), resulted in better CWP of M3 than M2. Next, the increase in the pore size of M2, M3 and M4 results in the improvement of water flow velocity due to the lower interaction of the water and the pore walls [35]. Although M2 has a larger pore size, the permeability is much more affected by the hydrophilicity rather than the pore size.

Figure 9 also shows that the CWP of M4 reduced to 169.64 L/(m^2^ h bar, which is lower than the CWP of M1 (181.55 L/(m^2^ h bar) and the M3 (800.6 L/(m^2^ h bar)). Despite having almost the same pore size as M3, the CWP of M4 is much lower than the CWP of M3. To explain these findings, we suspect a swelling effect on the M4 (see result in Table 4) due to a large amount of residual TA/PVA in the membrane matrix compounded with less hydrophilicity of M4 (Figure 9). Upon permeating, water wets the hydrophilic sites in the membrane matrix, swells them and renders the flow path of water to be smaller, resulting in a lower permeability being produced, which is proved by the percentage of water uptake summarized in Table 4. Alternatively, as observed from Figure 3, the pore structure with large macrovoids posed by M4 is likely to undergo severe compaction due to the pressure applied in the feed side, thus diminishing the CWP as mentioned elsewhere [36]. 

### 3.2. Hydraulic Performance

#### 3.2.1. Permeance Recovery

**Figure 11:**
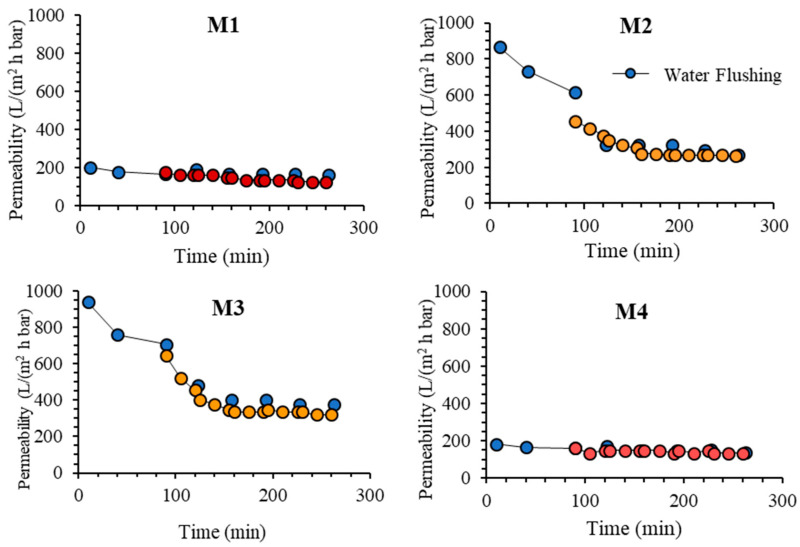
Permeability of oil/water emulsion filtration operated in cycles with intermittent water flushing.

Figure 11 shows the oil/water emulsion permeability of each membrane during five filtration cycles. The CWP after one hour of compaction is considered as stable and was used as a reference to evaluate the loss of permeability due to membrane fouling. After the compaction, the structure is assumed to be the same because the filtration tests were run at the same pressure [37]. In the oil/water emulsion filtrations, M3 also shows the highest final permeability of 540.18 L/(m^2^ h bar) compared to 165.18, 415.18 and 147.32 L/(m^2^ h bar), respectively for M1, M2 and M4. The great permeability increment of M2 and M3 compared to M1 supports the hypothesis proposed earlier on the increasing hydrophilicity of both membranes that assists in limiting the fouling effect. The hydrophilic membrane surface reduces the amount of oil particles from blocking the membrane pores which caused the membrane fouling [10]. Formation of hydrogen bonding between water molecules and the polar group on the membrane surface formed a monolayer water that limits the access of the oil droplets to the membrane surface and its interaction with it [32].

The results on the five cycles of oil/water emulsion filtrations in Figure 11 show that M3 outperforms the rest. Each membrane shows a reduction in permeability over time due to fouling and is only partly recovered by flushing. The result also shows that M4 poses the most stable permeability in which the permeability is almost constant over the entire filtration tests. Conversely, M1 shows a significant decline in permeability due to its hydrophobic properties. The significantly low permeability of M4 in comparison to M2 and M3 can be attributed to the swelling effect in the matrix of M4 where the hydrophilic components strongly interact with water that constricts or squeezes the pores. For such membrane, intrinsic fouling in the form of droplet entrapment in the membrane matrix can be avoided, resulting in excellent antifouling properties [12]. However, by considering the throughput, M3 is selected as the best performing membrane because it poses the highest steady state permeability. It shows 20% improvement when compared to M2. 

Overall, the finding on the oil/water emulsion filtration tests demonstrates the advantage of complexation in enhancing the hydraulic performance of the resulting membrane. Nevertheless, the degree of complexation must be optimized to maintain high permeability and avoid detrimental effects due to swelling. 

#### 3.2.2. Oil Rejection

**Figure 12:**
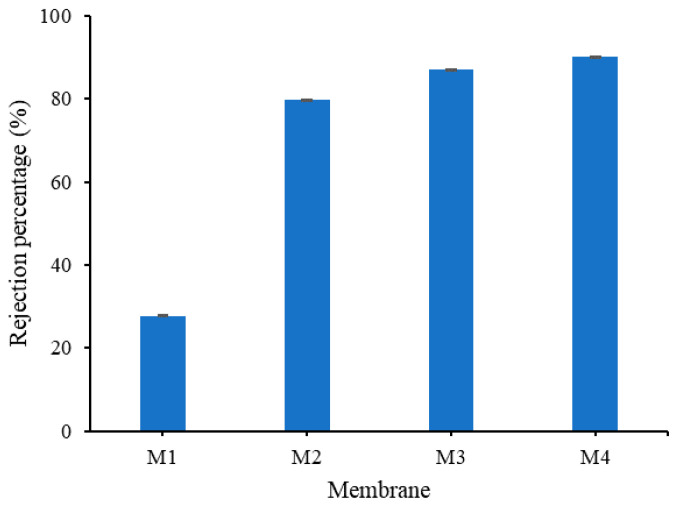
Oil rejection obtained from filtration of oil/water emulsion.

Figure 12 shows the oil/water rejection performance of all prepared membranes. All membranes containing TA and PVP (M2, M3 and M4) show excellent oil rejection performances. This implies that the pore size of the membrane is smaller than the average pore size of the oil particles in the emulsion and is also due to their improved hydrophilicity [23]. These modified membranes present >50% higher oil rejection than the pristine PVDF membrane (M1). 

M4 shows oil rejection of 90%, the highest among the membrane samples. The high rejection can be attributed to the internal hydrophilicity of M4 that restricts the flow of oil across the membrane matrix despite showing rather high WCA. It can be seen that the high hydrophilicity inside the structure offers advantages in rejection, but the swelling effect depresses the permeability. As a result of the hydrophilic properties which reside in the inner membrane matrix, more oil particles are rejected from flowing through the membrane. On the contrary, the hydrophobic property of the membrane matrix in M1 can be attributed to its poor oil rejection, despite having the smallest pore size [10]. 

#### 3.2.3. Fouling Resistance Analysis

**Figure 13:**
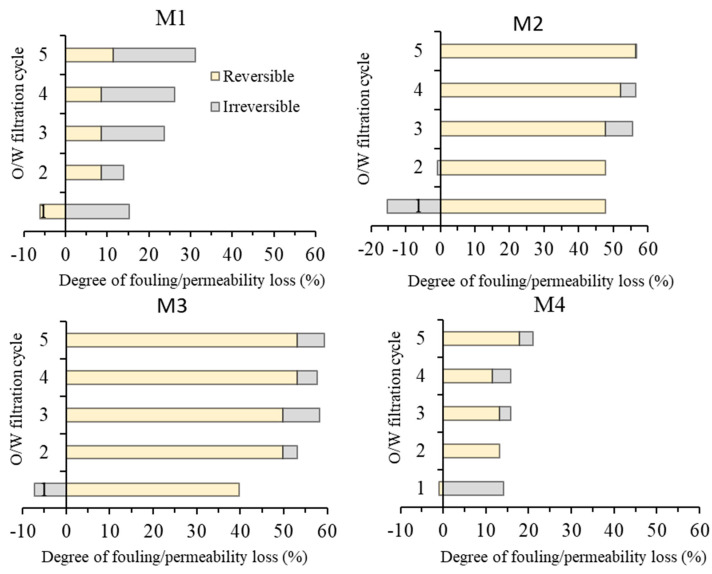
Fouling resistance analysis during five cycles of oil/water emulsion filtrations.

The oil/water emulsion filtration data suggest the advantage of M3 over the others in terms of permeability. To study the fouling properties, Figure 13 provides detailed analysis of the foulant cleanability, which was performed by identifying the irreversible and the reversible fouling of each membrane. The permeability lost from irreversible fouling can be recovered back through water flushing while irreversible fouling requires the usage of chemical cleaning [12]. 

A clear distinction can be made for low permeability (M1 and M4) and high permeability (M2 and M3). The magnitude of the permeability loss is in accordance with the oil/water emulsion permeability (in Figure 11). The comparison is discussed for the two pairs of the membranes. The pair of M1 and M4 are in the low-fouling regime (up to 30% of total fouling) due to their low permeability, while the pair of M2 and M3 is in the high fouling regime (of up to 60% total fouling). High permeability leading to high fouling because of the foulant was dragged by the flow of the permeate toward the membrane surface [38].

Both M1 and M4 show low overall fouling largely thanks to their low permeability. This epitomizes a flux-driven fouling concept. Nonetheless, M1 shows more fouling propensity than M4. M1 reaches total fouling of 30% at the fifth cycle, while M4 reaches the total fouling of 25% at the same stage. The high membrane fouling propensity for the M1 is due to its hydrophobic properties, which results in oil droplets/particles strongly interacting with the membrane and blocking the membrane pore, thus eventually reducing the permeability produced [10]. M4 shows an excellent antifouling property where after the first cycle, it only suffers a small percentage of irreversible fouling and is maintained until the last cycle. This supports the argument where hydrophilic additives are strongly preserved in the membrane matrix due to the complex formed with Fe^3+^ as suggested by the literature [33]. 

Both M2 and M3 are vulnerable to membrane fouling as their total fouling rates reach about 60%, in which M3 is slightly more prone to fouling, despite having better hydrophilicity. The slightly higher membrane fouling rate on M3 can be attributed to its higher water flux, in which the flow of water permeating through the membrane pore drags the oil droplets to the pore mouths to foul the membrane. Despite showing slightly higher total fouling rate, the final permeability of M3 of 361 L/(m^2^ h bar) is significantly higher than M2 of 261 L/(m^2^ h bar), much higher than what was achieved using the traditional additive (PVP and PEG) in our earlier reports [12,14]. The membrane fouling issue suffered by the membranes can further be addressed by optimizing operational parameters. Several operational approaches can be considered, to name a few, improved bubbling [39], imposing surface patterning [40], employing enhanced module or spacer system [41,42], or rotating spacer [43]. 

## 4. Conclusions

This study demonstrates the effectiveness of in situ formation of TA/PVP assembly aided by Fe^3+^ in enhancing the resulting membrane properties and oil/water filtration performance of PVDF-based membranes. The assembly of TA/PVP/Fe^3+^ could be formed during the phase inversion, which limits the degree of additive leaching. TA and PVP additives accompanied by Fe^3+^ solution in the non-solvent largely affect the resulting membranes’ characteristics. Dosing TA and PVP leads to the formation of membranes with large pores size (M2 = 1.54 vs. M1 = 0.22 µm) while addition of Fe^3+^ solution in the non-solvent lowers the pore size (M3 = M4 = 0.9 µm). The assembly of TA-PVP complexation was achieved as observed from the change in colour during the phase inversion as also universally proven from the FTIR spectra, XPS and EDX analysis. Incorporation of TA/PVP assembly leads to enhanced surface hydrophilicity by lowering the contact angle from 82° for M1 to 47° for M3. In situ assembly of the TA-PVP complex also leads to enhanced clean water permeability by a factor of four from M1 of 181.54 to M3 of 800.60 L/(m^2^ h bar) as a result of enhanced mean flow pore size from 0.2 to 0.9 µm and improved hydrophilicity. Owing to the enhanced surface chemistry and structural advantages, the optimum hydrophilic PVDF-TA-PVP membrane (M3) poses permeability of 540.18 L/(m^2^ h bar) and oil rejection of 87% for oil/water emulsion filtration, three times and twice the permeability and rejection of the pristine PVDF membrane (M1), respectively. The findings on the fouling rate suggests that the overall performance can still be enhanced because the optimum membrane (M3) still suffers a 60% loss from its intrinsic potential. 

## Figures and Tables

**Table 1:** Materials and weight percentage of all membrane samples.

Membrane Code	Dope Solution Composition (wt%)				Non-Solvent
	PVDF	TA	PVP	DMAC	
M1	15	-	-	85	Water
M2	15	7	1	77	Water
M3	15	7	1	77	0.001M Fe^3+^ solution
M4	15	7	1	77	0.05M Fe^3+^ solution

**Table 2:** Elemental composition of each membrane obtained by Energy Dispersive X-ray spectroscopy on the surface of SEM sample.

Membrane		Relative Composition (%)		
	Carbon	Fluorine	Nitrogen	Oxygen
M1	55.5	42.8	0.0	1.6
M2	53.6	40.2	0.0	5.7
M3	53.7	41.1	0.0	5.2
M4	52.3	42.6	0.0	5.1

**Table 3:** Summary of elemental concentration from XPS analysis.

Membrane		Atomic Concentration (%)		
	Carbon	Fluorine	Nitrogen	Oxygen
M2	13.10 ± 0.27	28.10 ± 0.31	2.72 ± 0.56	13.10 ± 0.27
M3	18.66 ± 0.29	20.02 ± 0.26	3.77 ± 0.58	18.66 ± 0.29

**Table 4:** The water uptake and swelling degree of the resulting membranes.

Membrane	Water Uptake (%)	Swelling Degree (%)
M1	20.18	6.38
M2	109.57	14.30
M3	81.82	8.44
M4	148.99	21.58

## Data Availability

The data presented in this study are available on request from the corresponding author.
